# Use of serum long non-coding RNA expression panel as a marker for diabetic retinopathy

**DOI:** 10.3389/fcvm.2025.1523997

**Published:** 2025-04-09

**Authors:** Eric Wang, Shali Chen, Anorin Ali, Biao Feng, Selina Liu, John Gonder, Tom Sheidow, Phil Hooper, Subrata Chakrabarti

**Affiliations:** ^1^ Department of Pathology and Laboratory Medicine, Western University, London, ON, Canada; ^2^ Centre for Diabetes, Endocrinology, and Metabolism, St. Joseph’s Hospital, London, ON, Canada; ^3^ Ivey Eye Institute, St. Joseph’s Hospital, London, ON, Canada; ^4^ Department of Pathology and Laboratory Medicine, London Health Sciences Centre, London, ON, Canada

**Keywords:** long non-coding RNA, diabetic retinopathy, epigenetics, diagnosis, biomarker

## Abstract

**Introduction:**

Diabetic retinopathy (DR) is the most common chronic complication of diabetes, the leading cause of vision impairments in working-aged adults, and a significant cause of reduced quality of life for diabetic patients. Diabetic patients are recommended to have regular screening in order to catch DR at an early enough stage for effective management. However, due to a variety of factors, many patients can still fall through the cracks with the current screening methods.

**Methods:**

Several long non-coding RNAs (lncRNAs), essential regulators of physiological and pathological processes, were previously identified by us as potential markers for DR phenotypes. In this study, we used a significantly larger sample set to validate our panel of lncRNAs. We also explored the possibility of creating a statistical model to detect DR from serum samples using the expression profiles of these lncRNAs.

**Results:**

Our regression models, based solely on lncRNA expression data, demonstrated the ability to adequately detect DR and potentially predict it. Models based solely on lncRNA expression performed equally or better compared to models with additional patient information. The models showed promising performance, suggesting that serum lncRNA expression profiles could serve as reliable markers for DR detection.

**Discussion:**

Further longitudinal studies are necessary to validate the model's capability to predict retinopathy in diabetic patients not yet diagnosed with DR. Nevertheless, our findings indicate that this lncRNA panel may offer a viable option for a simple, accessible, and convenient blood-based screening test for DR.

## Introduction

Diabetes and its complications pose significant challenges to patients’ qualities of life (1, 2). Diabetic retinopathy (DR) is among the most common chronic complications of diabetes and is the leading cause of vision impairment in working-aged adults (3, 4). In 2020, DR was responsible for over 4.3 million visually impaired patients in the world (5). Nearly all diabetic patients will develop retinopathy over the course of their lifetime, and a significant portion will develop vision impairments (6, 7). Patients with diabetes experience progressive hyperglycemic damage to the retinal tissues, and undergo progressive stages of retinopathy, starting from no retinopathy to non-proliferative DR (NPDR), and ultimately to proliferative DR (PDR) (8–11). Clinically, each stage of DR manifests as ophthalmologically distinct findings, ranging from microaneurysms and microhemorrhages in early NPDR to neovascularization and vitreous/preretinal hemorrhage in PDR (8–11). Some studies have estimated that ∼18% of patients with baseline NPDR progress to severe NPDR or PDR within 5 years of diagnosis (12). During this progression, diabetic macular edema—which can occur in either NPDR or PDR—and neovascularization in PDR remain major factors leading to blindness (8–11).

Despite its high prevalence in patients with diabetes, the progression of DR can be influenced by many factors. Poor glycemic control, dyslipidemia, hypertension, high body mass index, and a variety of genetic and epigenetic factors can all influence the progression from non-vision threatening to vision threatening DR (8–11, 13). Given the multifactorial nature of DR progression, early diagnosis and intervention can be vital in preventing vision impairment in patients with diabetes (14). Over the past several decades, regular ophthalmic screening and timely treatment has helped reduce the frequency of vision impairment due to DR (15), yet the current approaches to DR screening are not without their limitations. Screening and diagnosis of DR are currently done via ophthalmologic techniques such as ophthalmoscopy, fluorescein angiography, and optical coherence tomography, depending on their availability at specific practices. These approaches rely on clinical observations of physical manifestations of retinal changes (such as visible vascular abnormalities and thickening of retinal membranes) (16, 17). Such changes are the result of accumulated hyperglycemia-induced biochemical and cellular abnormalities, which can be detected long before observable manifestations (18). Furthermore, socioeconomic factors such as cost, accessibility, and convenience mean that many patients do not adhere to the recommendations for vision care and screening (19–21). Clinical access to instruments/tools, the nature of the screening approaches, patient access to healthcare, and other socioeconomic factors limit the benefits conferred by regular ophthalmological examinations. A simple, standardized, blood-based test may help improve ease of access by being administered when patients get their regular bloodwork, and timeliness of diagnosis by detecting underlying biochemical changes. This would ultimately improve ophthalmic outcomes for diabetic patients.

As with most chronic diseases, molecular and biochemical alterations both underlie and precede clinical manifestations in DR, for example, altered expressions of extracellular matrix genes underpin the changes in basement membrane thickness which lead to retinal vascular leakage (22). We have previously identified changes in specific long non-coding RNAs (lncRNAs) associated with the onset and progression of DR (23). lncRNAs are RNA molecules greater than 200 bases in length, which do not encode proteins (23). lncRNAs exert influences on gene expression through a variety of means, and can regulate a wide range of biological processes and are involved in the pathogenesis of various diseases (24–26). Pertinently, lncRNAs are known to regulate pathways including angiogenesis, inflammation, and endothelial-to-mesenchymal transition, all of which are important in the pathogenesis of DR (27–29). We previously investigated the development of a serum-based, multi-panel PCR test using 9 lncRNAs (ANRIL, MALAT1, WISPER, ZFAS1, H19, HOTAIR, HULC, MEG3, and MIAT) as a potential diagnostic and prognostic tool (23). With the relatively limited patients that were recruited for that study, we found that a substantial portion of patients with some forms or retinopathy could be identified using only the panel of lncRNAs or some subset thereof (23). We believe based on our previous findings, that the lncRNA panel can be improved for use as a blood test for the diagnosis of DR. Here, we used a refined panel of lncRNAs in an expanded patient population to validate the development of our PCR-based screening and diagnostic test for DR.

## Materials and methods

### Patient population and sample collection

This study was approved by the Western Research Ethics Board and Lawson Health Research Institute at the University of Western Ontario (London, ON, CAN). Informed consent was received from patients prior to obtaining specimens and the research was conducted in accordance with the principles outlined by the *Declaration of Helsinki*.

Patients were recruited from endocrinology or ophthalmology clinics in London ON (*n* = 317). The clinicians approached consecutive patients under their care as appropriate using broad eligibility criteria (see below). All patients with diabetes and diabetic retinopathy were considered for enrollment, no other known diabetic complications or symptoms of other diseases were present. In parallel, non-diabetic patients were also similarly approached. Eligible patients were approached by a member of the team, who introduced them to the study. If the patient expressed interest, a member of the research team gave them necessary literature and explained the study in greater detail. Written, informed consents were obtained. All patients’ charts were reviewed for data collection including age, sex, duration of diabetes, type of diabetes etc. Table 1 outlines inclusion and exclusion criteria. All samples were collected between 2022 and 2023 during office visits, then immediately transported to the lab for processing. Demographic information about the patients can be found in Table 2. 

**Table 1:** Inclusion/exclusion criteria.

Inclusion criteria		Exclusion criteria
Diabetic group	Non-diabetic group	
• ≥18 years of age • Has been clinically diagnosed with diabetes mellitus (type I or type II)	• ≥18 years of age • No history of diabetes	• Pregnant or breastfeeding • Enrolled in another randomized controlled trial that could influence the outcomes or data collection of this study • Unable to provide written consent

**Table 2:** Demographic data.

Demographic parameter	Non-diabetic controls (ND)	All diabetic patients (D)	Diabetic patients without retinopathy (D-NR)	Diabetic patients with retinopathy (DR)	Retinopathy patients with NPDR (NPDR)	Retinopathy patients with PDR (PDR)
Sample size	55	262	97	165	87	78
Age	49.16 ± 17.33	63.15 ± 15.59	62.94 ± 19.18	63.28 ± 13.08	65.93 ± 12.11	60.32 ± 13.57
Sex (F:M)	26:29	115:147	45:52	70:95	32:55	38:40
Duration of diabetes (years)	NA	20.42 ± 11.76	19.42 ± 12.92	20.99 ± 11.04	18.6 ± 12.92	23.7 ± 11.65

Data are expressed as mean ± SD where applicable; DR encompasses NPDR and PDR groups; D encompasses D-NR and DR groups. F, female; M, male.

Undiluted serum samples were collected in BD gold-top serum separator tubes. Serum specimens received a code number and were submitted to the research laboratory. All assessments were performed in a masked fashion.

### Development of the multi-lncRNA qPCR panel

We developed a multi-lncRNA qPCR panel based on 8 lncRNAs of interest as investigated in the previous study (23), namely, ANRIL, H19, HOTAIR, HULC, MALAT1, MIAT, WISPER, and ZFAS1; MEG3, which was examined previously had been excluded because it did not show clear enough differences in disease progression. Customized exon-spanning primers against human lncRNAs were designed as described previously. Primers for our lncRNAs of interest, and the reference gene ACTB, were air-dried in a 96-well plastic qPCR plate and stored at room temperature. cDNA was synthesized from patient serum samples and mixed with TB green master mix (Takara Bio, Mountain View, CA, USA) and aliquoted into the 96-well PCR plates containing the dried primer panel. lncRNA expressions were quantified using a LightCycler 96 system (Roche Diagnostics, Laval, QC, CAN).

### RNA isolation and quantitative real-time polymerase chain reaction (Rt-qPCR)

Total RNA was isolated from 200 μl of serum using TRIzol reagent (Invitrogen) as previously described (23). RNA concentration was measured using a SpectraMax QuickDrop Spectrophotometer (Molecular Devices); RNA purity was also determined via OD 260/280 readings. Most samples had ratios greater than 1.9, no samples had 260/280 ratios lower than 1.7. cDNA was synthesized using 2 μg of total RNA with the High-Capacity cDNA Reverse Transcription kit (Applied Biosystems, Catalog #: 4368813) according to the manufacturer's instructions. qPCR was performed using a Roche LightCycler 96 system (Roche) as described previously (23). qPCR results were retrieved using the LightCycler 96 SW 1.1 software (Roche) and expression levels were calculated using the standard curve method. β-actin was used as an internal control for sample normalization (e.g., to account for potential differences in cDNA synthesis due to varying RNA purity readings). All qPCRs were performed in triplicates.

### Data preprocessing and outlier removal

Distributions of MALAT1 and ZFAS1 expressions were highly right skewed, so their levels were log-transformed to better capture the patterns of changes in expression, and outliers were removed from the dataset. Using a variation of the “boxplot rule”, outliers were classified as samples with two or more lncRNA expression levels above 3rd quartile plus 1.5 times the interquartile range or below 1st quartile minus 1.5 times the interquartile range.

### Clinical group comparisons

*T*-tests were used to compare between two clinical groups. For comparisons involving more than two groups, ANOVA tests were used. If the ANOVA results indicated statistical significance, pairwise *t*-tests were performed with Holm adjustment for multiple comparisons. Significance was considered at *p* < 0.05 throughout the analysis.

### Binomial regression for diabetic retinopathy

Binomial regression was conducted with diagnosis of DR of diabetic patients as the response variable and lncRNA expression levels as predictive factors. The resulting mathematical equation estimates a subject's likelihood—expressed as a probability between 0.0 and 1.0—of having diabetic retinopathy. To assess multicollinearity of regressors and prevent errors such as overfitting, Pearson correlation coefficient (PCC) statistic was utilized. In the case that 2 or more lncRNAs were highly correlated, the weaker predictor was removed.

### Cross-validation and optimal cut-off point

The dataset was randomly split into 80% training data and 20% testing data for cross-validation. The optimal cut-off point was determined using a receiver-operating characteristic (ROC) curve and Youden's index. The ROC curve plots sensitivity against the false-positive rate (1−specificity) over a range of cut-off values. The best cut-off point for defining a positive test was identified using Youden's index: Youden's index = max(true positive rate—false positive rate). At this point, significant improvements in sensitivity were achieved while maintaining reasonable specificity. The cross-validated accuracy, specificity, and sensitivity were calculated at the optimal cut-off point and reported along with the area under the curve (AUC). The significance of the variables in the final model was assessed by 95% confidence intervals.

### Statistical analysis

All analyses were conducted in RStudio (Version 2022.07). The rstatix package was used to perform all statistical tests comparing clinical groups. Ggplot2, ggcorrplot, and ggpubr were used for data visualization.

## Results

### Serum lncRNA expressions are altered in patients with diabetes

**Figure 1:**
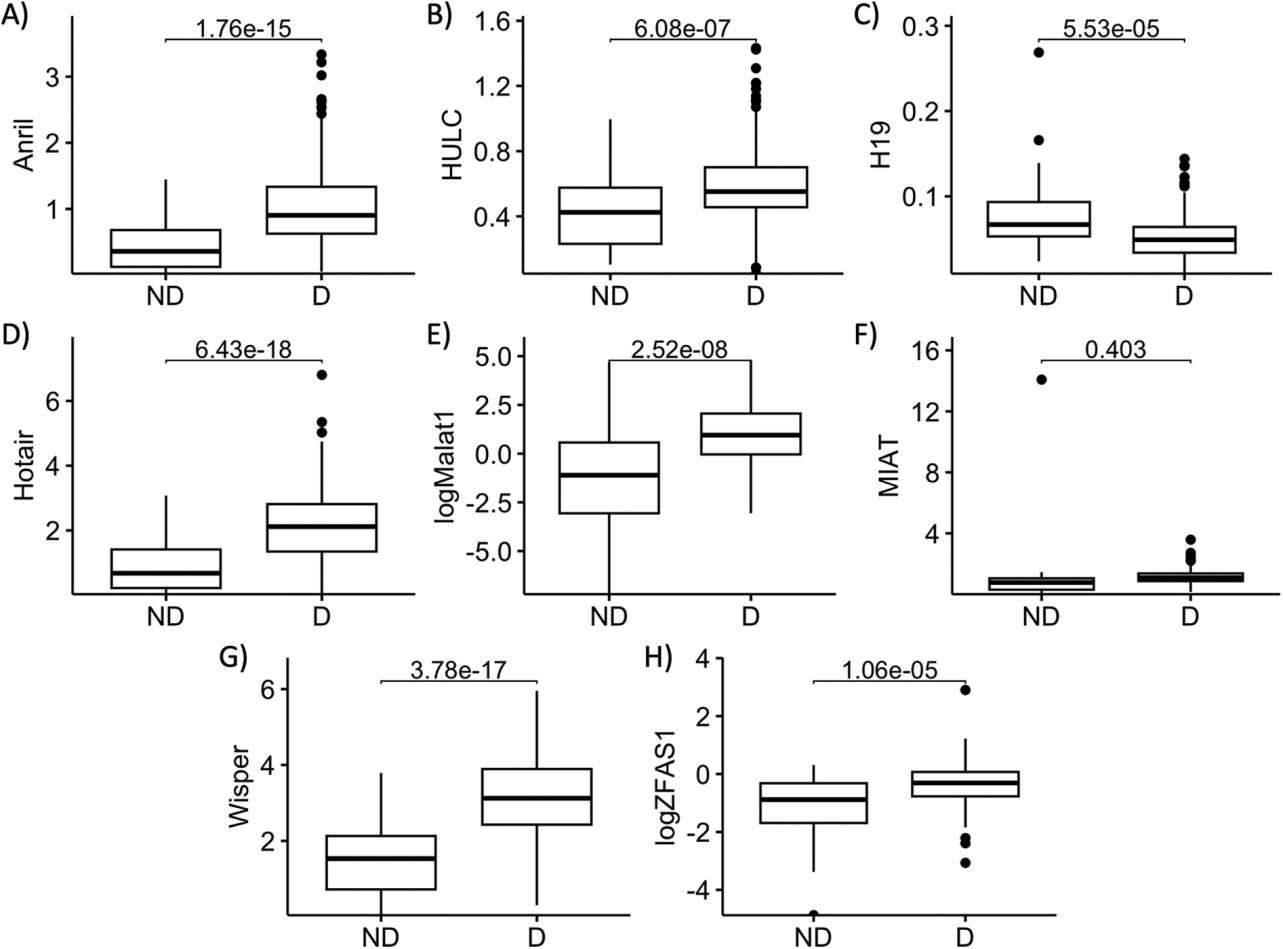
Serum abundances of key lncRNAs are different between diabetic and non-diabetic patients. Serum levels of **(A)** ANRIL, **(B)** HULC, **(C)** H19, **(D)** HOTAIR, **(E)** MALAT1, **(F)** MIAT, **(G)** WISPER, and **(H)** ZFAS1 in non-diabetic (ND) and diabetic (D) patient serums were compared. Apart from MIAT, serum levels of all other lncRNAs of interest were significantly different between D and ND patients. lncRNA data presented as ratio to ACTB mRNA. MALAT1 and ZFAS1 data were log-transformed to normalize the distributions. Statistical significances were calculated using Student's *T*-test. *n* = 55 for ND, and *n* = 262 for D groups.

We first assessed the changes in expressions of individual lncRNAs between the diabetic and the non-diabetic patients. We conducted a correlation analysis between patients’ ages and lncRNA expressions—as there was a significant difference in ages between the ND and D patients—and found no significant correlations between age and any of the lncRNA expressions via the Pearson test. In accordance with our previous findings, serum expressions of ANRIL, HOTAIR, HULC, MALAT1, WISPER, and ZFAS1 were significantly higher in the D compared to the ND group, while expression of H19 was significantly lower (Figures 1A–E,G,H). We found no significant difference in the serum expression of MIAT between D and ND patients in our sample (Figure 1F), so it was therefore dropped from subsequent analyses.

### Serum lncRNA expressions show specific patterns in diabetic patients at different stages of Dr

**Figure 2:**
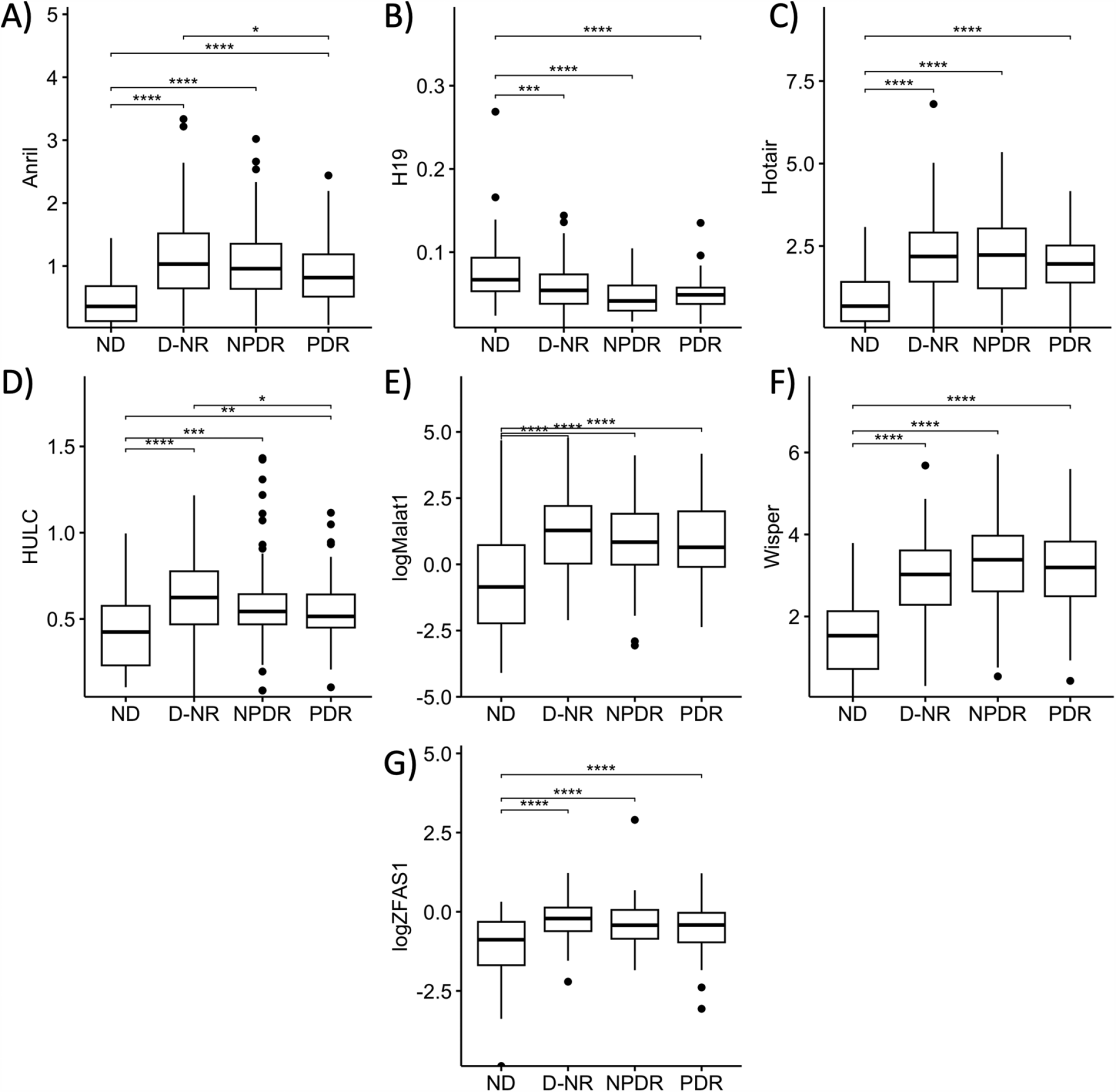
Serum abundances of lncRNAs of interest vary between stages of diabetes. Comparison of serum levels of **(A)** ANRIL, **(B)** HULC, **(C)** H19, **(D)** HOTAIR, **(E)** MALAT1, **(F)** WISPER, and **(G)** ZFAS1 across non-diabetic (ND), diabetic without retinopathy (D-NR), non-proliferative diabetic retinopathy (NPDR) and proliferative diabetic retinopathy (PDR) groups. Data showed that on average, derangements in serum lncRNA abundance were most significant in D-NR or NPDR groups, and that lncRNA levels recovered slightly in the PDR group compared to the D-NR group. lncRNA data presented as ratio to ACTB mRNA. MALAT1 and ZFAS1 data were log-transformed to normalize the distributions. Statistical significances were calculated using one-way ANOVA and *post-hoc t*-tests were performed with Holm's adjustment. *n* = 55 for ND, *n* = 97 for D-NR, *n* = 87 for NPDR, and *n* = 78 for PDR groups. *p < 0.05, **p < 0.01, ***p < 0.001, or ****p < 0.0001.

Having established diabetes-related changes in serum expressions of our lncRNAs of interest, we took a closer look at differential lncRNA expressions in diabetic patients at differing stages of DR. To this extent, we broadly categorized the diabetic patients into 3 categories: D-NR, NPDR, and PDR. Apart from MIAT, expressions of lncRNAs in D-NR, NPDR and PDR groups were all significantly different from the non-diabetic group. Interestingly, the data showed on average, that altered lncRNA expressions peaked in the D-NR or NPDR groups and showed a slight return to normal levels in PDR patients. Expressions of ANRIL and HULC in the PDR cohort was significantly lower than in the D-NR group (Figures 2A,D). Expressions of H19, HOTAIR, MALAT1, WISPER and ZFAS1 also showed slight trends back toward levels seen previous stages in the PDR groups (Figures 2B,C,E–G).

### Serum lncRNA expressions can be used to identify diabetic patients with DR

**Figure 3:**
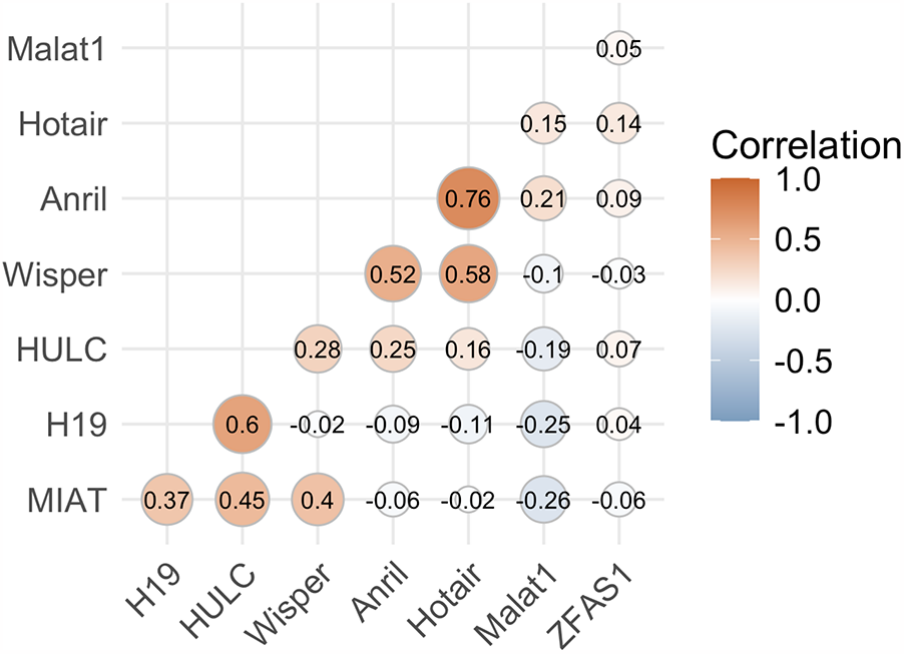
lncRNA ANRIL correlates strongly with lncRNA HOTAIR. Correlation analysis was performed to assess and prevent multicollinearity in the regressors. ANRIL and HOTAIR were strongly correlated above our cutoff of ±0.75, so HOTAIR was ultimately dropped from the regression model to prevent overfitting and other errors.

**Figure 4:**
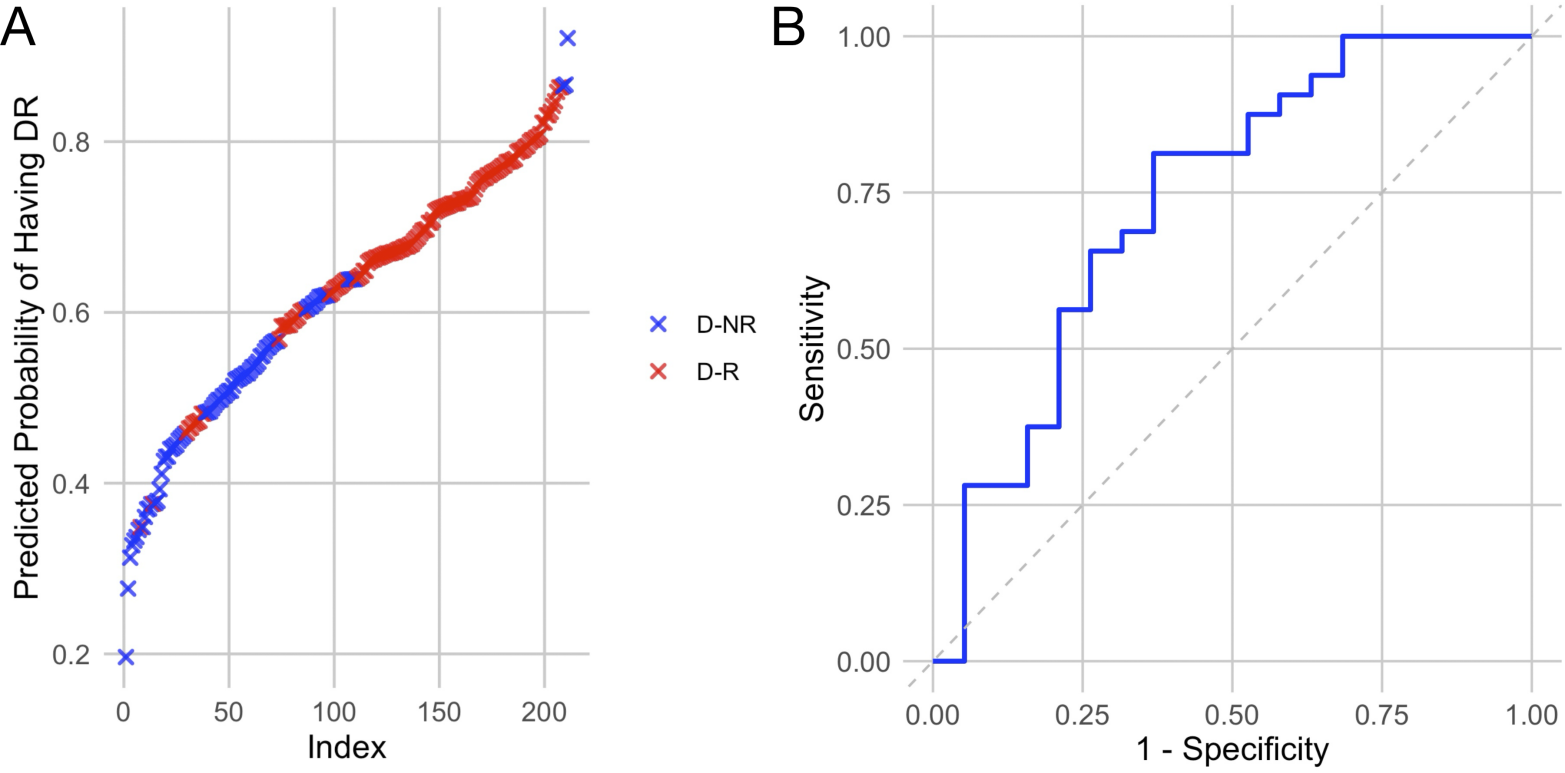
Regression model built using lncRNAs of interest largely separates patients with retinopathy from those without. **(A)** Regression plot showing predicted likelihood of patients having or not having retinopathy based on their lncRNA expression profiles. **(B)** Receiver operating characteristic curve showing the sensitivity and specificity of the model. Based on lncRNA expressions, the model predicted that some diabetic patients without retinopathy are likely to have retinopathy, resulting in a slightly lower specificity.

Assessment of average lncRNA expressions across stages of DR allowed us to identify trends in the sample populations but were insufficient to predict DR in individual diabetic patients. To assess the likelihood of retinopathy for individual patients, we conducted binomial regression using lncRNA expressions as predictive factors. Because ANRIL and HOTAIR were strongly correlated, HOTAIR was removed from binomial regression (Figure 3). For this analysis, we grouped the NPDR and PDR cohorts into a DR cohort. The regression plot showed relatively clean separation of the no DR and DR groups (Figure 4A). For diabetic patients, this model achieved a predictive accuracy of 75% with respect to presence or absence of retinopathy, with a sensitivity of 81%, a specificity of 63%, and an AUC of 0.74 (Figure 4B). As a point of reference, we built a model based only on patient demographic information, including age, sex, and duration of diabetes. This model performed poorly, with an AUC of 0.55, and almost indiscriminately predicted positive DR status in all diabetic patients (Table 3). Combining demographic data with lncRNA expression data did not necessarily improve the predictive performance of the model (Table 3), furthermore patient age and duration of diabetes were the lowest ranked coefficients in the model's algorithm (not shown).

**Table 3:** Performance metrics for models.

Model performance parameter	Demographic data only (Age + sex + duration)	lncRNA only	lncRNA + Demographic data^a^
Accuracy	0.666	0.745	0.745
Sensitivity	1	0.8125	0.625
Specificity	0.105	0.632	0.947
AUC	0.546	0.737	0.786

^a^ This model reduces the significance of lncRNAs as predictive factors and shows signs of overfitting, which can be common in models with more predictive factors.

### Glycated hemoglobin levels did not improve the predictive performance of our model compared to lncRNA only

Glycemic control typically has more influence on the development of retinopathy than simple demographic metrics. We wanted to assess whether glycemic control information—in the form of HbA1c levels—could improve the performance of the model. Due to limitations with data access and data collection, HbA1C data was only available in a smaller subset of patients (*n* = 75), so we re-trained our other models as described above, using only datapoints from patients with HbA1c data fairer comparison. With this smaller dataset, the base model using only demographic data performed similarly (Table 4). Adding HbA1C improved the accuracy but reduced the AUC (Table 4). The accuracy of the lncRNA only model was better in this subset of patients than in the larger sample, and the addition of HbA1c did not produce major differences (Table 4). We further tested a model using all the components, which performed worse than lncRNA only (Table 4). Duration of diabetes and patient age remain the two lowest ranking coefficients in the model's algorithm (not shown).

**Table 4:** Model performances only including patients with HbA1c data.

Model performance parameter	Demographic data only	Demographic data + HbA1C	lncRNA only	lncRNA + HbA1C	lncRNA + HbA1c + Demographic data^a^
Accuracy	0.643	0.714	0.857	0.857	0.786
Sensitivity	1	0.833	0.667	0.667	0.833
Specificity	0.375	0.625	1	1	0.75
AUC	0.604	0.5625	0.833	0.8125	0.75

^a^ This model reduces the significance of lncRNAs as predictive factors and shows signs of overfitting, which can be common in models with more predictive factors.

## Discussion

Diabetes has long since reached pandemic levels, and recent projections suggest a doubling of the prevalence of diabetes by 2050 (30). Such a rise will inevitably give rise to increased incidences of DR and other diabetic complications. DR is the most common microvascular complication of diabetes, affecting an estimated 27% of patients with diabetes (3, 31). Data have shown that early detection and intervention in DR have played a vital role in achieving better ophthalmic outcomes for patients over the past several decades (15). Yet current screening approaches are limited by various factors (16–21). We set out to create an affordable biomarker-based screening test for diabetic retinopathy which can bypass the limitations of current approaches. We have previously described a panel of lncRNAs which change in response to retinopathy in diabetic patients (23). In the present study, we validated our previous findings and refined the lncRNA panel using a larger sample size and with patients from all stages of DR (D-NR, NPDR, PDR). We then reported that lncRNA derangement appears to progressively increase as diabetic patients progressed from D-NR to NPDR, but that some lncRNAs showed slight trends back to previous levels in PDR. We further determined that we were able to adequately predict retinopathy using 7 of the original 9 lncRNAs. Finally, we compared our lncRNA-based model against models built using other patient metrics and assessed the potential of combining lncRNA expression with said metrics to build a better prediction model.

LncRNAs are considered master regulators of cellular processes. One lncRNA, by interacting with various proteins and other species of RNAs, can exert a broad range of effects on gene expression and cellular behaviour. The lncRNAs of interest for this test—namely ANRIL, H19, HOTAIR, HULC, MALAT1, WISPER, and ZFAS1—were initially identified in other pathologies, as evident in some of their names, **h**ighly **u**pregulated in **l**iver **c**ancer (HULC) (32), or **m**etastasis **a**ssociated **l**ung **a**denocarcinoma **t**ranscript 1 (MALAT1) (27). But these lncRNAs have since been well-studied in the context of DR. For example, we have shown in DR, that ANRIL regulates VEGF (33, 34), H19 inhibits inflammatory responses and prevents endothelial dysfunction (28), HOTAIR promotes angiogenesis, oxidative stress and mitochondrial dysfunction (29), and MALAT1 regulates inflammation via modulation of epigenetic regulators (27). Others have also reported on the roles of these lncRNAs in DR (35–40), thus we felt that it may be feasible to use integrate expression profiles of these lncRNAs to create a diagnostic tool. Because lncRNAs are upstream regulators of many processes, a lncRNA-based diagnostic tool would be able to detect a much earlier stage in the development of DR than current optical screening approaches. Furthermore, we believe that a lncRNA-based test can begin detecting DR before other emerging screening tools, such as deep-learning approaches which are still dependent on the presence of vascular changes (41), and even other biomarkers which examine changes in abundances of proteins (42), metabolites (43), or microRNAs (44), all of which typically occur downstream of lncRNA expression changes. We began with a larger panel of lncRNAs in our initial pilot study and refined it through the results of that study and the present one. lncRNAs with high variation and/or low changes between ND and D groups were dropped from the test, the remainder were further analyzed and assessed for the creation of the predictive model.

For lncRNAs that showed significant alterations between ND and D groups, the D group was broken down into D-NR, NPDR, and PDR. This allowed use to identify patterns in the lncRNA expressions throughout the progressive stages of DR, and to build expression profiles for diabetic patients without DR and those with DR. Given that many studies have shown aberrations in lncRNA expression to contribute to the pathogenesis of DR, we had expected to see progressively greater deviance from normal expressions as patients progressed from D-NR to PDR. This was interestingly, not the case. Expressions of many lncRNAs showed a slight reversal in PDR patients. The underlying cause of this reversion is not within the scope of the current study; however, it may be logical to expect that the treatments received by PDR patients may play some role in this finding. Treatments in PDR are designed to prevent and potentially reverse neovascularization and vascular leakage, thus it may be the case that patients who receive treatment for their PDR also see a mild reversal in their lncRNA expressions with the amelioration of their conditions. This trend may otherwise be explained by the pathobiology of DR's disease progression. As the early stages of DR are considered vaso-obliterative, while the PDR stage is considered vaso-proliferative, the genetic and biochemical changes underpinning these phases may be different (8–11). However, as the sample size of present study is not large enough to control for specific treatment among PDR patients, any proposed reason behind this phenomenon is merely speculative. Further in-depth studies will be required to fully elucidate the mechanistic reasons behind this trend and explore the effects of PDR treatments such as laser photocoagulation and vitrectomy on lncRNA expressions. It is important to note that while some lncRNA expressions in PDR trended to similar levels as D-NR, they are still significantly different from the ND group. Furthermore, this analysis showed that there is relatively little difference in expressions of individual lncRNAs between the diabetic sub-populations compared to the difference in expressions between diabetic and non-diabetic groups. In addition, from a technical standpoint, use of more sensitive techniques, such as nano-PCR may potentially increase the sensitivity of these assays. Such notion however must be validated by additional well- designed studies.

While individual lncRNA expressions did not clearly separate between different sub-groups within the diabetic condition, each of the stages did have a distinct pattern of lncRNA expressions, thus we were optimistic that together, the lncRNAs could be used to build a model to better predict DR. To this extent, we opted to group the patients into D-NR and DR, because we did not think it would be useful to develop a model that can specifically predict PDR, as that would not offer any benefits over currently available tests. The model achieved a predictive accuracy of 75% and an AUC of 0.745, compared with a model built only from patient demographic data, which only achieved an accuracy of 66% and an AUC of 0.546. The demographic data-only model serves as a baseline, because the development and progression of DR are dependent on various factors in addition to demographic characteristics of the patient. We further tried using different combinations and subsets of lncRNAs to train the model, but those failed to match the main model. The lncRNA model had a higher sensitivity (81%) than specificity (63%) meaning that it had a slight tendency toward false positive predictions. This is in contrast with a model built using both demographic data and lncRNA, which had a similar predictive accuracy and AUC as the lncRNA-only model but had a much higher specificity (94.7%) than sensitivity (62.5%). We believe that high sensitivity is the key performance metric for the models, as it means that the model can correctly identify DR in patients who have it. We further think that a relatively lower specificity could be a promising result, because if the model is predicting that a patient from the D-NR group has a high likelihood of DR, it may be that this patient has undiagnosed DR, or is close to developing clinically detectable DR. This claim is something that must be assessed over a longer study period with follow-up, we cannot assess this claim in given the current study parameters, however, given the promising results, we are optimistic that a longitudinal study would show the benefits of the model.

Although we had promising results from the lncRNA-only model, we still wondered if mixing it with other factors could lead to better accuracy. One of the most important factors in the development of diabetic complications is glycemic control (45). Poor glycemic control leads to average higher glucose levels and accelerated tissue damage in diabetes. Differences in glycemic control may account for why patient age and duration of diabetes were consistently the worst performing coefficients in our models, as an older patient with longer duration of diabetes but good glycemic control may have better ocular health than a younger patient with poor glycemic control. It is widely accepted that patients with poor glycemic control will develop complications faster than one with good glycemic control (45). So, we wanted to see if including HbA1C levels, a widely used indicator of glycemic control, would benefit the predictive accuracy of the model. As mentioned, only a smaller subset of patients had HbA1C data available, so all the models were re-trained on this smaller sample size. Unsurprisingly, demographic data alone did not produce a good model. Demographic data with the addition of HbA1C showed an improvement compared with demographic data without HbA1C. However, the lncRNA models with or without HbA1C performed similarly, and much better than the two previously mentioned models. This finding was initially surprising, however, further review of the literature showed that while extremely high HbA1C levels correspond to elevated risk for progression of complications, HbA1C levels in the moderate to high range may not correlate as strongly with disease progression (46, 47). Furthermore, retinopathy can be triggered by sharp decreases in systemic glucose and HbA1C levels (48–50), and genetic variation can influence how HbA1C levels correlate with glycemic control (51, 52). Furthermore, HbA1C is a risk factor for DR, while the lncRNA expression panel acts as a biomarker, which are more directly correlated with DR progression. This means that HbA1C may not be a good addition to the model to identify patients with DR, and that the model built with only lncRNA expression data likely remains the best option.

While we firmly believe that the findings of the current study are promising, we are cognizant of the limitations of the study and its parameters. All samples were collected from centers around London Ontario, meaning that there is limited demographic diversity, thus potentially limiting the generalizability of the findings. Furthermore, despite all sample collections being done in London Ontario, there were variations in the times between collection and processing/analysis which can introduce unwanted variability due to the potential for RNA to degrade. Separately, we recognize the limitations of using HbA1C levels as a representation of glycemic control, because though it is more reliable than fasting glucose, is also subject to variation depending on short- to medium-term glycemic control and lifestyle changes. Furthermore, HbA1C data was not available from all patients, and although available data were split evenly between diabetic patients with or without DR, available data for distinct subgroups of DR were limited. We also note that a longitudinal study would be required in order to properly validate the notion that the lncRNA-based model can detect D-NR patients who are at high risk for DR or who have undiagnosed DR. We are planning such a longitudinal study with patients being recruited from a wider background.

Regular ophthalmic screening and timely treatment have greatly benefitted diabetic patients over the past decades (15). However, current screening approaches are limited by factors such as their need for clinical observations of physical changes and low patient adherence to recommended screening, meaning that many diabetic patients can develop DR and be undiagnosed for a long period of time, negatively influencing the timeliness of effective treatment, leading to poorer ophthalmic outcomes. The current study proposes a promising serum-based biomarker test which can bypass these limitations. The proposed serum lncRNA-based panel is based on lncRNAs that are involved in the pathobiology of DR, and are significantly altered in response to diabetes. Expression profiles of these lncRNAs can be integrated to predict DR in diabetic patients with 74.5% accuracy against clinically diagnosed cases. Further validation, including longitudinal studies and multi-centre patient recruitment, will be needed to fully develop this test, however, the final panel should be able to detect DR at a stage prior to the onset of physical vascular manifestations and can be done in tandem with regular blood tests, leading to greater accessibility and adherence to DR screening, and resulting in better ophthalmic outcomes for patients with diabetes.

## Data Availability

The raw data supporting the conclusions of this article will be made available by the authors, without undue reservation.
